# Supplementary material for: Development of a computer-aided design software for the quantitative evaluation of aesthetic damage
Source: PLoS One. 2019 Dec 18;14(12):e0226322. doi: 10.1371/journal.pone.0226322 (PMC6919621; doi:10.1371/journal.pone.0226322)
Supplement: S1 Code site — 1. (ZIP) [file pone.0226322.s001.zip › [EESC_jr][Projeto]Relatorio_07_21/reports/templates_zip/gentelella-master/production/form_advanced.html]

Gentellela Alela! | 


Gentellela Alela!

Welcome,

## John Doe

  


### General

- Home 
  - Dashboard
  - Dashboard2
  - Dashboard3
- Forms 
  - General Form
  - Advanced Components
  - Form Validation
  - Form Wizard
  - Form Upload
  - Form Buttons
- UI Elements 
  - General Elements
  - Media Gallery
  - Typography
  - Icons
  - Glyphicons
  - Widgets
  - Invoice
  - Inbox
  - Calendar
- Tables 
  - Tables
  - Table Dynamic
- Data Presentation 
  - Chart JS
  - Chart JS2
  - Moris JS
  - ECharts
  - Other Charts
- Layouts 
  - Fixed Sidebar
  - Fixed Footer

### Live On

- Additional Pages 
  - E-commerce
  - Projects
  - Project Detail
  - Contacts
  - Profile
- Extras 
  - 403 Error
  - 404 Error
  - 500 Error
  - Plain Page
  - Login Page
  - Pricing Tables
- Multilevel Menu 
  - Level One- Level One
      - Level Two
      - Level Two
      - Level Two
    - Level One
- Landing Page Coming Soon

- John Doe
  - Profile
  - 50%
    Settings
  - Help
  - Log Out
- 6
  - John Smith
    3 mins ago

    Film festivals used to be do-or-die moments for movie makers. They were where...
  - John Smith
    3 mins ago

    Film festivals used to be do-or-die moments for movie makers. They were where...
  - John Smith
    3 mins ago

    Film festivals used to be do-or-die moments for movie makers. They were where...
  - John Smith
    3 mins ago

    Film festivals used to be do-or-die moments for movie makers. They were where...
  - **See All Alerts**


### Form advanced

Go!

## Input Mask

- - Settings 1
  - Settings 2

Date Mask

Phone mask

Custom Mask

Serial Number

TaxID Mask

Credit Card Mask

Cancel
Submit


## Color Picker

- - Settings 1
  - Settings 2

Default Input

Styled

Horizontal bar

Vertical bar

Inline picker


## Input knob

- - Settings 1
  - Settings 2

Display value

× 'cursor' mode

Step 0.1

Angle arc

Alternate design

Angle offset

## Date Pickers Available with multiple designs

- - Settings 1
  - Settings 2

December 30, 2014 - January 28, 2015

Date Range Picker with opening to right and left

December 30, 2014 - January 28, 2015

|  | Mar 2013 | | | | |  |
| --- | --- | --- | --- | --- | --- | --- |
| Su | Mo | Tu | We | Th | Fr | Sa |
| 24 | 25 | 26 | 27 | 28 | 1 | 2 |
| 3 | 4 | 5 | 6 | 7 | 8 | 9 |
| 10 | 11 | 12 | 13 | 14 | 15 | 16 |
| 17 | 18 | 19 | 20 | 21 | 22 | 23 |
| 24 | 25 | 26 | 27 | 28 | 29 | 30 |
| 31 | 1 | 2 | 3 | 4 | 5 | 6 |

|  | Mar 2013 | | | | |  |
| --- | --- | --- | --- | --- | --- | --- |
| Su | Mo | Tu | We | Th | Fr | Sa |
| 24 | 25 | 26 | 27 | 28 | 1 | 2 |
| 3 | 4 | 5 | 6 | 7 | 8 | 9 |
| 10 | 11 | 12 | 13 | 14 | 15 | 16 |
| 17 | 18 | 19 | 20 | 21 | 22 | 23 |
| 24 | 25 | 26 | 27 | 28 | 29 | 30 |
| 31 | 1 | 2 | 3 | 4 | 5 | 6 |

From

To

Apply 
Cancel

(success)

|  | Mar 2013 | | | | |  |
| --- | --- | --- | --- | --- | --- | --- |
| Su | Mo | Tu | We | Th | Fr | Sa |
| 24 | 25 | 26 | 27 | 28 | 1 | 2 |
| 3 | 4 | 5 | 6 | 7 | 8 | 9 |
| 10 | 11 | 12 | 13 | 14 | 15 | 16 |
| 17 | 18 | 19 | 20 | 21 | 22 | 23 |
| 24 | 25 | 26 | 27 | 28 | 29 | 30 |
| 31 | 1 | 2 | 3 | 4 | 5 | 6 |

|  | Mar 2013 | | | | |  |
| --- | --- | --- | --- | --- | --- | --- |
| Su | Mo | Tu | We | Th | Fr | Sa |
| 24 | 25 | 26 | 27 | 28 | 1 | 2 |
| 3 | 4 | 5 | 6 | 7 | 8 | 9 |
| 10 | 11 | 12 | 13 | 14 | 15 | 16 |
| 17 | 18 | 19 | 20 | 21 | 22 | 23 |
| 24 | 25 | 26 | 27 | 28 | 29 | 30 |
| 31 | 1 | 2 | 3 | 4 | 5 | 6 |

From

To

Apply 
Cancel

(success)

|  | Mar 2013 | | | | |  |
| --- | --- | --- | --- | --- | --- | --- |
| Su | Mo | Tu | We | Th | Fr | Sa |
| 24 | 25 | 26 | 27 | 28 | 1 | 2 |
| 3 | 4 | 5 | 6 | 7 | 8 | 9 |
| 10 | 11 | 12 | 13 | 14 | 15 | 16 |
| 17 | 18 | 19 | 20 | 21 | 22 | 23 |
| 24 | 25 | 26 | 27 | 28 | 29 | 30 |
| 31 | 1 | 2 | 3 | 4 | 5 | 6 |

|  | Mar 2013 | | | | |  |
| --- | --- | --- | --- | --- | --- | --- |
| Su | Mo | Tu | We | Th | Fr | Sa |
| 24 | 25 | 26 | 27 | 28 | 1 | 2 |
| 3 | 4 | 5 | 6 | 7 | 8 | 9 |
| 10 | 11 | 12 | 13 | 14 | 15 | 16 |
| 17 | 18 | 19 | 20 | 21 | 22 | 23 |
| 24 | 25 | 26 | 27 | 28 | 29 | 30 |
| 31 | 1 | 2 | 3 | 4 | 5 | 6 |

From

To

Apply 
Cancel

(success)

|  | Mar 2013 | | | | |  |
| --- | --- | --- | --- | --- | --- | --- |
| Su | Mo | Tu | We | Th | Fr | Sa |
| 24 | 25 | 26 | 27 | 28 | 1 | 2 |
| 3 | 4 | 5 | 6 | 7 | 8 | 9 |
| 10 | 11 | 12 | 13 | 14 | 15 | 16 |
| 17 | 18 | 19 | 20 | 21 | 22 | 23 |
| 24 | 25 | 26 | 27 | 28 | 29 | 30 |
| 31 | 1 | 2 | 3 | 4 | 5 | 6 |

|  | Mar 2013 | | | | |  |
| --- | --- | --- | --- | --- | --- | --- |
| Su | Mo | Tu | We | Th | Fr | Sa |
| 24 | 25 | 26 | 27 | 28 | 1 | 2 |
| 3 | 4 | 5 | 6 | 7 | 8 | 9 |
| 10 | 11 | 12 | 13 | 14 | 15 | 16 |
| 17 | 18 | 19 | 20 | 21 | 22 | 23 |
| 24 | 25 | 26 | 27 | 28 | 29 | 30 |
| 31 | 1 | 2 | 3 | 4 | 5 | 6 |

From

To

Apply 
Cancel

(success)


## Grid Slider

- - Settings 1
  - Settings 2

Default grid slider with min and max values

Grid with slider labels are far away outside it's container

Grid with labels inside container using force\_edges attribute

Create Grid with pre-defined steps

Prevent one from dragging the labels

Grid with minimum and maximum values

Grid With time in 24 hour format

  
  


X

px

Y

px

Width

px

Height

px

Rotate

deg

ScaleX

ScaleY

Get Cropped Canvas


160×90


320×180

×

#### Cropped

Close
Download


Get Data


Set Data


Get Container Data


Get Image Data


Get Canvas Data


Set Canvas Data


Get Crop Box Data


Set Crop Box Data


0,0


100%


180°

16:9


4:3


1:1


2:3


Free

VM0


VM1


VM2


VM3

Toggle Options

- responsive
- restore
- checkCrossOrigin
- checkOrientation
- modal
- guides
- center
- highlight
- background
- autoCrop
- movable
- rotatable
- scalable
- zoomable
- zoomOnTouch
- zoomOnWheel
- cropBoxMovable
- cropBoxResizable
- toggleDragModeOnDblclick

Cropper.js


Gentelella - Bootstrap Admin Template by Colorlib
